# False Dawns and New Horizons in Patient Safety Research and Practice

**DOI:** 10.15171/ijhpm.2017.115

**Published:** 2017-09-19

**Authors:** Russell Mannion, Jeffrey Braithwaite

**Affiliations:** ^1^ Health Services Management Centre, University of Birmingham, Birmingham, UK.; ^2^ Institute of Health Innovation, Macquarie University, Sydney, NSW, Australia.

**Keywords:** Health System, Patient Safety, Adverse Events, Preventable Harm, System Thinking

## Abstract

In response to a weight of evidence that patients are frequently harmed as a result of their care, there have been concerted efforts to make healthcare safer, with health systems across the globe investing significant resources in policies and programmes designed to reduce adverse events. Yet, despite extensive efforts, improvements in safety have proved difficult to sustain and spread, with studies confirming there has been no measurable, systems-level improvement in the overall rates of preventable harm. Here, we highlight the limitations of the thinking which underpins current efforts to make healthcare systems safer and point to new and emerging approaches to understanding and addressing patient safety in complex, dynamic health systems.

## Try Again. Fail Again. Fail Better ^[1]^

It has been recognised since antiquity that medical practice brings the possibility for both patient benefit as well as the potential for adverse clinical outcomes.^1^ This problematic is reflected in the guiding principle, *primum non nocere* (first do no harm) which remains a touchstone of contemporary codes of professional ethics. Nevertheless, history is replete with examples of where misguided but well-intentioned medical interventions have generated more harm than good^ [2] ^. The true scale of patient harm in health systems remained latent and under-recognised, and was not well known (even by the medical, nursing and allied professions) until comparatively recently.

Dating back to the early 1990s, successive international research studies that have assessed the extent of adverse events have accumulated evidence to suggest that around one in 10 patients experience some type of health-related harm while receiving inpatient care.^2^ Similar rates appear to hold in other health settings.^3^ The burgeoning field of patient safety research has highlighted failures of both commission (eg, medication errors) and of omission (eg, the failure to provide recommended care).^4-7^ In response to this mounting weight of evidence, there have been concerted efforts to make healthcare safer, with health systems across the globe investing significant resources in a bewildering array of policies and research programmes designed to reduce the burden of patient harm.^8,9^ As with many other aspects of health system reform, the medical profession has been a key influence in shaping research and policy in relation to patient harm, first by rejecting, then by gradually accepting, and finally by embracing the patient safety agenda.^10^

There have undoubtedly been gains in making care safer, for example, through decreased catheter-related blood stream infections,^11^ lower mortality and morbidity attributed to the use of checklists in operating theatres,^12^ and the widespread adoption of Medical Emergency Teams and Rapid Response Systems to deal with deteriorating patients.^13^ Yet despite extensive efforts by many committed and well-intentioned policy-makers, managers, clinicians, researchers and patient groups, it is disconcerting that improvements in safety have been confined to a few celebrated examples or niche areas such as these. Where there have been solutions advanced, they have proved difficult to sustain and spread, with recent studies confirming there has been little or no measurable improvement in the overall rates of preventable harm at the systems level.^2^ Indeed, amid growing recognition that most patient safety problems are not amenable to simple solutions, it is apparent that the original optimism of the patient safety movement has given way to hard bitten realism.^2,14^

## System Heal Thyself

Perhaps we have not been sufficiently sophisticated in specifying solutions to the problem of ameliorating harm. The system is complex and the problem is a wicked one.^15^ Linear, uni-dimensional solutions—let’s run a hand hygiene campaign, let’s issue more policy, let’s regulate more extensively, let’s mandate root cause analysis in all cases of severe harm—applied to care settings, have fallen short of expectations for them.^16^ That being the case, the question becomes, what models, studies or concepts lay better foundations for improvements in patient safety?

Current approaches have deep historical and institutional antecedents that have been affirmed over decades, and are woven into the mindsets of those involved in health services research, healthcare policy and professional practice. Traditionally, the dominant mode of thinking about patient safety was predicated on the assumption of individual culpability, with errors and adverse events mainly attributed to incompetence, negligence and individual personality deficits such as carelessness, forgetfulness or recklessness. Based on this logic, individual clinicians, so called ‘bad apples,’ were blamed and held personally accountable for any errors made. Even so, in practice the medical profession largely failed historically to address wrongdoing and failures against professional regulatory procedures focused on identifying and rooting out ‘bad apple’ miscreants. Famous cases such as those in which the GP Dr Harold Shipman, working in the English NHS, systematically murdered his patients, and instances of large-scale inquiries into extensive harm,^17,18^ highlight this.

In order to tackle things differently, since around the turn of the millennium, stimulated amongst other factors by cases such as these, patient safety has increasingly been viewed as a systems issue whereby errors and adverse events are thought to arise mainly from dysfunctions in the wider systems of care rather than as the consequences of personal fallibility.^19^ A new “safe systems orthodoxy” gradually emerged, in which errors and adverse events were attributed to aberrations in the “system.” In this framing, clinicians are not so much seen as culpable or reckless, but as interacting agents in problematic systems or cultures. Interventions were oriented towards designing working environments that anticipated and minimised the impact of human error, with a focus on reducing both ‘active failures,’ ie, mistakes and unsafe acts made by interacting health professionals working at the sharp end of care delivery, and addressing more upstream ‘latent failures’ located in the wider environment (eg, problematic organisational cultures, poor team dynamics, time pressures or deficits in workload scheduling). This kind of early systems thinking was underpinned by a conviction that reorganising formal structures, streamlining systems of care, or purposively managing organisational cultures would deliver the desired improvements in patient safety.^20^ This systems approach spawned many interventions built on a multiplicity of conceptualisations of the problem, including root cause analysis, the swiss cheese model, lean techniques, checklists, and the like.

## Fault Lines in Systems Thinking

But this systems approach, while an advance on earlier linear attempts to improve things, has not taken us sufficiently far. It has relied on what we think of as simplistic systems thinking. It is the version of systems theory that underpins many current efforts to make care safer, but has a number of serious limitations which may, in part, explain why progress has still been so painfully slow. First, this kind of systems thinking, which underpins the approach we now know as Safety-I, still assumes at its heart that adverse outcomes can be explained by linear cause-effect chains, as originally proposed by the Domino metaphor^21^ and embodied in the widespread use of patient safety frameworks and tools such as incident reporting and root cause analysis.^19,22^ Although it does bring into the safety arena a systems science perspective, this framing of errors and adverse events nevertheless sees that they have a definable cause which can be ‘found and fixed.’ It remains focused on locating the causes of failure, rooting out aberrations, and introducing interventions in an attempt to eliminate (or at least attenuate) their distal causes.

However, it is clear that the logic inherent in this approach no longer corresponds to the reality of care settings, if ever it did. The lack of success in improving patient safety over these last two decades may be due to the concepts and methods underpinning systems-oriented approaches not matching the profound complexity of modern healthcare settings. Care is delivered in intricate, fragmented, sometimes chaotic settings, in complex political, socio-cultural environments with a virtually infinite range of moving parts and interconnections. Healthcare is characterised by informalities, work-arounds, feedback loops, emergent behaviours, politics, nested networks, fractal properties, systems dynamics, and bottom-up adaptiveness.^23^ These kinds of settings stubbornly resist the introduction of top-down, standardised policy, regulations or linear-style interventions.^24,25^ They may even induce serious deleterious consequences if there are substantial mismatches between the putative solutions and their intended target.^26^

Another, related limitation of simplistic systems thinking is that the ‘system’ to be fixed is primarily conceived as the local ‘micro-system,’ comprising local clinical and team behaviours responsive to upper-echelon prescriptions, rather than more broad-based factors, such as profession-wide social structures and cultural norms.^27,28^ Indeed, a fatal flaw in the Safety-I approach is that it is heavily infused with thinking which has been described as work-as-imagined.^4^ Work-as-imagined is what those working at the blunt end of the health system (eg, policy makers, regulators, planners, directors and researchers) believe or think should happen at the sharp end of care delivery when they design improvement strategies or attempt to influence or nudge the behaviours of those working at the sharp end. Much of this is predicated on encouraging and mandating adherence to a multiplicity of rules, regulations and external performance metrics and standards. Work-as-done on the other hand is what people do on the front-lines of care to get the job accomplished in complex settings which are always very different from the way those remote from the front lines imagine them to be. Sometimes clinicians do provide care that corresponds to the ways those who envision and prescribe it at the blunt end imagine it to be, but this is rare. This is because people working at the sharp end do so in resource-constrained, challenging and culturally unique circumstances, and for the most part they accomplish their tasks by employing informalities, localised patchwork-quilt solutions, and work-arounds.^29,30^ In short, clinicians on the front lines of care flex and adjust their practices to fit with, and map to, the local contexts, demands, contingencies and cultural characteristics, rather than in response to top-down regulation, policies and standard operating procedures.

Despite the on-the-ground variation, complexities, and local adjustments in clinical care, things go right far more often than they go wrong. This is a key insight of those advocating Safety-II.^4,14,28,31,32^ This argues that a feature of clinical work that has been paid too little attention is that it succeeds far more often than it fails. As a consequence of this insight and argument, in such complex adaptive systems, we need to better understand how work is actually accomplished across different healthcare settings and thereby seek to become more accomplished at matching work-as-imagined and work-as-done. This would mean we need to get better at designing work-as-imagined policies, regulations and standards so that they much closer align to, and reflect an enhanced understanding of, how work is actually done.^33^ It would also mean we need a system in which those at the sharp end of care delivery have a better appreciation of what is being sought by those doing work-as-imagined, and an enhanced appreciation of their own organisation cultures and systems.^20^ Yet in health systems research, culture and culture change are often poorly theorised and based on the overly simplistic assumption that cultures are malleable and can be readily manipulated and managed to beneficial effect.^34,35^ We should be cautious, too, in drawing parallels between healthcare and other industries, such as aviation, as it is problematic to import ideas wholesale from very different cultural contexts and expect them to be taken up unproblematically.^36^

In short, most systems improvement strategies to date have been based on imagining work rather than how it is done in practice.^4^ That is to say, those at the apex of the hierarchy have determined, funded or prescribed policies or initiatives to be embraced by those on the front lines of clinical care without adequate understanding of the complex intricacies and nuances inherent in the delivery of modern healthcare.

## New Approaches and Future Directions for Research

As we have seen, progress in making care safer for patients has been sufficiently slow such that the momentum is seen to have stalled. The rates of harm appear to have flatlined at 10%. This necessarily calls into question the dominant ways in which patient safety has been framed and addressed. Albert Einstein is credited with the familiar aphorism, *“Insanity is doing the same thing over and over again and expecting different results.”* If we want to achieve different results then we need to be less reverential towards the orthodox paradigm, get beyond simplistic system thinking, expand our research horizons, and advance new and better ways for understanding and intervening in patient safety. One approach is to take the thinking originating from resilience engineering, the Safety-II approaches identified above, and begin to focus more clearly on how things typically go right. This is a radically wide-ranging view of the system. It argues that the same behaviours produce good care as produce poor care, and seeks to understand the complex health system in more comprehensive ways. This extended view of safety argues that attention must be placed on the conditions under which people succeed rather than fail, instead of looking myopically at why things go wrong.

The Safety-II perspective recognises that both failures and successes have their origin in performance variability (at both the individual and systemic levels), and that it is equally mistaken to attribute successes to careful planning and diligence as it is to attribute failures to individual incompetence or error. An important practical implication flows from this argument. We cannot appreciate a system and its performance by looking solely or predominantly at harm. We must direct attention to the whole system, and the conditions under which it operates, if we are to make gains in understanding. We will not apprehend the system in all its complexity by looking only at abnormalities when healthcare delivery fails.

Complementing this approach, we point to other recent work drawing on complexity science applied to healthcare.^32,37-41^ This calls into view the dynamic nature of the system, the relationships that deliver care, and its interactional characteristics. We also point to advances and evolving research from across the social sciences such as recent studies on the sociology of patient safety,^10^ including new dramaturgical perspectives on the organisational governance of patient safety,^42^ increasingly persuasive research on the potential for patient and relatives to contribute towards safer care,^43^ and the use of (behavioural) economic levers and incentives to motivate organisations and individuals to provide safer care for patients.^8^

Taken together, these new horizons work with the complex dynamics of healthcare instead of assuming that things will change by instrumental means, by operationalising top-down linear solutions. They also show, much more profoundly than previously, how superficial conceptualisations of systems will not do. We need to form deep knowledge of the nature of the healthcare complex adaptive system, its resilient expressions and capacities, and mobilise a wider range of stakeholders including patients, to the patient safety enterprise.

That is the new horizon, and we see glimpses of evidence, reported here, that we are shifting toward it. There are grounds to believe, at least for the optimists among us, as we have argued above, that it will not be yet another false dawn. But only time will tell whether, and the extent to which, we can make more tangible progress in our journey, reaching toward this new horizon.

## Ethical issues

Not applicable.

## Competing interests

Authors declare that they have no competing interests.

## Authors’ contributions

Both authors contributed equally to the writing of this article.

## Authors’ affiliations

^1^Health Services Management Centre, University of Birmingham, Birmingham, UK. ^2^Institute of Health Innovation, Macquarie University, Sydney, NSW, Australia.

## Endnotes


[1] Becket S. Worstward Ho. New York, NY: Grove Press Inc; 1983.

[2] Classic examples include the use of mercury and arsenic as medicines in the 18th Century and Thalidomide in the 1960s.

